# Sexual dimorphism in an animal model of Sjögren's syndrome: a potential role for Th17 cells

**DOI:** 10.1242/bio.013771

**Published:** 2015-10-09

**Authors:** Alexandria Voigt, Lida Esfandiary, Cuong Q. Nguyen

**Affiliations:** 1 Department of Infectious Diseases and Pathology, College of Veterinary Medicine, University of Florida, 2015 SW 16th Ave, Gainesville, FL 32608, USA; 2 Center for Orphan Autoimmune Disorders, University of Florida College of Dentistry, 1600 SW Archer Rd, Gainesville, FL 32608, USA

**Keywords:** Sjögren's syndrome, Sexual dimorphism, Th17 cells, Animal model

## Abstract

Sjögren's syndrome is a complex autoimmune disease with an array of diverse immunological, genetic and environmental etiologies, making identification of the precise autoimmune mechanism difficult to define. One of the most distinctive aspects of Sjögren's syndrome is the high sexual dimorphism with women affected 10-20 times more than men. It is nearly impossible to study the sexual dimorphic development of Sjögren's syndrome in human patients; therefore it is pertinent to develop an appropriate animal model which resembles human disease. The data indicated that female C57BL/6.NOD-*Aec1Aec2* mice developed an earlier onset of sialadenitis with a higher composition of CD3^+^ T cells and a 10-fold increase in glandular infiltration of Th17 cells at the onset of clinical disease compared to male mice. Inflammatory Th17 cells of female mice exhibited a stronger proliferation in response to disease-specific antigen than their male counterpart. At the clinical disease stage, altered autoantibody patterns can be detected in females whereas they are seldom observed in male C57BL/6.NOD-*Aec1Aec2* mice. Interestingly, male C57BL/6.NOD-*Aec1Aec2* mice developed an earlier loss of secretory function, despite the fact that female C57BL/6.NOD-*Aec1Aec2* mice exhibited a more rapid secretory loss. This data indicates the strong sexual dimorphism in the SjS-susceptible C57BL/6.NOD-*Aec1Aec2* animal model, making it an appropriate animal model to examine human disease.

## Abstract (precis)

**Summary:** Sjogren's syndrome (SjS) is the one of the most common sexual dimorphic autoimmune diseases. This study demonstrates that female C57BL/6.NOD-*Aec1Aec2* mice develop earlier onset and more severe symptoms of SjS than male counterparts. Therefore, the C57BL/6.NOD-*Aec1Aec2* mouse model could be an appropriate model to study sexual dimorphism in SjS.

## INTRODUCTION

Sjögren's syndrome (SjS) is a chronic, systemic autoimmune disease characterized most notably by development of dry eyes and dry mouth manifestations, indicative of secretory dysfunction of the lacrimal and salivary glands (Bolstad and Jonsson, 2002; Chiorini et al., 2009; Delaleu et al., 2005; Jonsson et al., 2004). The pathogenesis of SjS reveals a complex and heterogeneous array of diverse immunological, genetic and environmental phenotypes, making identification of the precise autoimmune mechanism(s) difficult to define. It is emerging as one of the most common systemic autoimmune human diseases, affecting primarily post-menopausal women with the onset usually between 40-60 years of age (Fox and Kang, 1992; Nguyen and Peck, 2009; Sullivan, 1997). It has one of the highest sexual dimorphisms among autoimmune diseases with women affected 10-20 times more than men (Nikolov and Illei, 2009; Porola et al., 2007; Sullivan, 1997). This high prevalence in women might suggest hormonal involvement, perhaps an imbalance between estrogen and androgen (Sullivan, 1997, 2004; Toda et al., 1998). SjS affects females usually in middle-age or post-menopausal stages, a time when estrogen levels are drastically decreased (Burger et al., 2002). This observation is rather counterintuitive since an increased level of estrogen is positively correlated with other autoimmune diseases such as systemic lupus erythematosus (SLE) and rheumatoid arthritis (RA) (Pennell et al., 2012). Interestingly, in addition to the decline in estrogen levels, androgen is also decreased due to the gradual retreat of dehydroepiandrosterone (DHEA) secretion by the adrenal glands (Valtysdottir et al., 2001). Therefore, temporal changes in levels of estrogen and androgen during menopause might contribute to the initiation of SjS in female patients, yet the precise mechanism(s) that govern the dimorphic autoimmune process involving these sex hormones remains unknown.

Sexual dimorphism in SjS has been observed in animal models. In the NOD.H2^h4^ model, females exhibited more severe sialadenitis with a higher number of CD4^+^ T cell infiltration in the salivary glands and elevated levels of autoantibodies compared to males (Čiháková et al., 2009). The comparison of different strains of mice by Toda et al. (1999) determined that inflammation was more predominant in the lacrimal and salivary glands of female MRL/lpr, MRL+, F1, C3H/lpr and gld mice compared to males. Lacrimalitis is significantly more severe in male non-obese diabetic (NOD) mice while severe sialadenitis was observed in female NOD mice. Therefore, depending on the mouse strains, dimorphic disease development can be observed in different exocrine tissues. Using the SjS-susceptible (SjS^s^) NOD and C57BL/6.NOD-*Aec1Aec2* mouse models, we have postulated that the development of SjS progresses through three distinct, but continuous phases (Lavoie et al., 2011; Nguyen and Peck, 2009). The initial phase starts with the glandular pathology and a number of aberrant genetic, physiological and biochemical activities, accompanied by the adaptive immune phase associated with migration of leukocytes expressing pro-inflammatory cytokines to the exocrine glands. The unregulated inflammation in the glands induces bystander cell death, which initiates a pro-inflammatory milieu, thereby perpetuating the on-going glandular damage by macrophages and natural killer (NK) cells. Differential gene expression analysis demonstrated the involvement of IFN-γ and IFN-γ signature genes, which activate specific chemokines and adhesion molecules, particularly VCAM and E-cadherin, allowing for the influx of inflammatory cells in the glands (Peck and Nguyen, 2012; Peck et al., 2011). Consequently, the loss of secretory functions occur establishing the clinical phenotypes of SjS, most likely the result of antagonistic autoantibodies reacting with the muscarinic receptor type III (M3Rs) (Bacman et al., 2001; Nguyen et al., 2000; Sumida et al., 2012). These phases define an innate inflammatory response, followed by an adaptive autoimmune response. The molecular events that occur at each phase can be modulated differently by sex hormones, for example salivary gland epithelial cells from SjS patients downregulated IFNγ-induced activation of ICAM.1 when treated with 17β-estradiol (E2) (Manoussakis et al., 2012). Estrogen plays a significant role in regulating both innate and adaptive immune cells, e.g. enhancing antigen presentation of dendritic cells, activation of pathogenic T helper 17 (Th17) cells and affecting autoantibody secretion by B cells (Pennell et al., 2012). Androgen has an opposite effect on Th1 cell differentiation by inhibiting IL-12-induced Stat4 signaling of CD4+ T cells (Kissick et al., 2014).

The multifactorial functions of sex hormones with the complexities of the autoimmune process hinder a comprehensive understanding of sexual dimorphic etiology in SjS in human patients. The goal of this study was to examine the sexual dimorphism exhibited by the murine mouse model using an established SjS^s^ animal model, C57BL/6.NOD-*Aec1Aec2*, in which the disease development is defined by temporal specific patho-immunological events (Brayer et al., 2000; Cha et al., 2002a). We have found an earlier onset of sialadenitis with a higher composition of CD3+ T cells and Th17 cells in females compared to males. Salivary gland cells of female mice exhibited stronger proliferative potential than males. At the clinical disease stage, altered autoantibody patterns can be detected in females whereas they are minimally observed in male mice. Interestingly, male C57BL/6.NOD-*Aec1Aec2* mice developed an earlier loss of secretory function; however, female C57BL/6.NOD-*Aec1Aec2* mice exhibited a more rapid secretory loss. These data indicate the strong sexual dimorphism in SjS^s^ C57BL/6.NOD-*Aec1Aec2* animal models, making it an appropriate animal model to examine human disease.

## RESULTS

### Severe influx of salivary gland lymphocytes initiated during the adaptive phase of Sjögren's syndrome in female C57BL/6.NOD-Aec1Aec2 mice

**Fig. 1.**
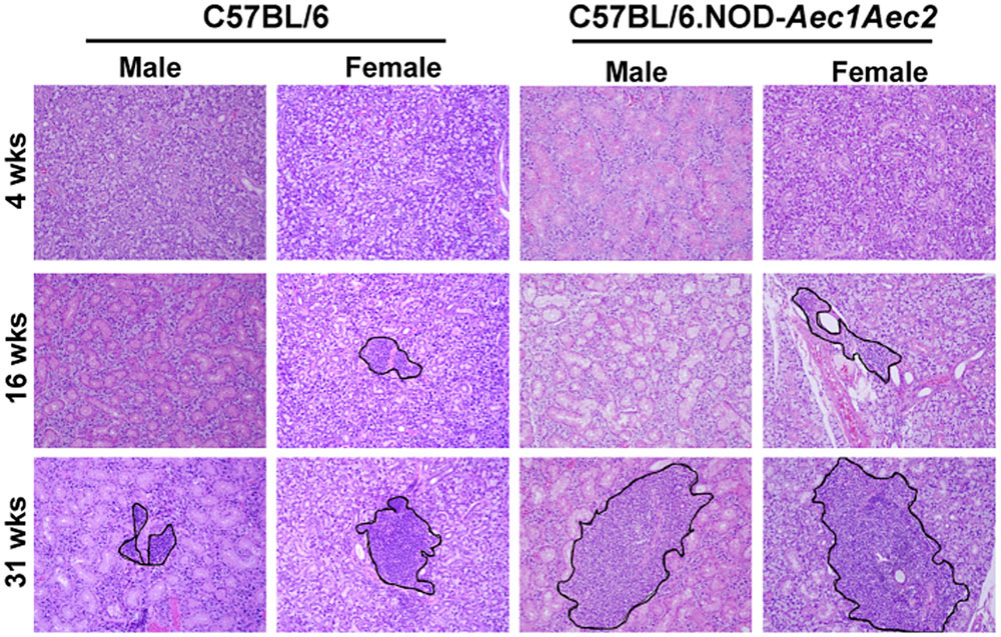
**Temporal lymphocytic infiltrations in salivary glands.** Salivary glands of male and female C57BL/6 and C57BL/6.NOD-*Aec1Aec2* mice (*n*=6/sex/strain) were excised at 4 weeks, 16 weeks and 31 weeks of age. Glands were placed in 10% phosphate-buffered formalin for 24 h. Fixed tissues were embedded in paraffin and sectioned at a thickness of 5 μm. Paraffin-embedded sections were deparaffinized by immersing in xylene, followed by dehydration in ethanol and H&E staining. Representative images were presented at 200× magnification with lymphocytic infiltrates traced in black.

**Fig. 2.**
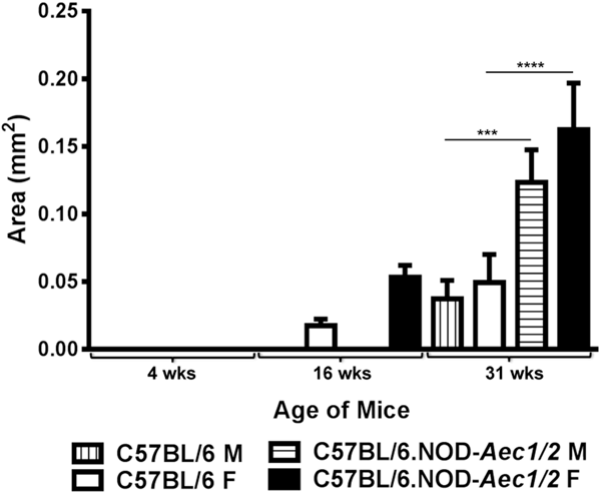
**Severity of sialadenitis in salivary glands.** Glands of male and female C57BL/6 and C57BL/6.NOD-*Aec1Aec2* mice (*n*=6/sex/strain) were excised at 4 weeks, 16 weeks and 31 weeks of age. Based on the H&E staining presented in previous figure, area of sialadenitis was calculated using Nikon Elements software with region of interest (ROI) function via a trace around the infiltrate. Statistical significance was obtained using two-way ANOVA. Error bars indicate s.e.m. with ***P<0.001, ****P<0.0001.

Previous studies have demonstrated that aberrant pathophysiological changes are detected in 4 weeks old C57BL/6.NOD-*Aec1Aec2* mice, followed by lymphocytic infiltration at approximately 16-20 weeks of age in male and female C57BL/6.NOD-*Aec1Aec2* mice (Brayer et al., 2000; Cha et al., 2002a). Starting from 24 weeks of age, both males and females developed secretory dysfunction with full penetrance. Our previous studies have demonstrated that sialadenitis precedes the loss of secretion in both male and female of C57BL/6.NOD-*Aec1Aec2* mice, however it is unknown how sialadenitis is observed differently and temporally differentiated in both sexes. In order to compare the inflammatory lesions in the salivary glands, we have used male and female C57BL/6.NOD-*Aec1Aec2* mice with sex- and age-matched C57BL/6 controls in this study to examine the changes in infiltration in the glands over the course of the disease. As presented in Fig. 1, at a pre-disease stage of 4 weeks of age, no noticeable inflammation was observed in C57BL/6.NOD-*Aec1Aec2* or C57BL/6 mice. During the adaptive phase of the disease, 16 weeks (wks) of age, female C57BL/6.NOD-*Aec1Aec2* mice began to develop lymphocytic foci, while male C57BL/6.NOD-*Aec1Aec2* mice showed little or no infiltration at a similar age. Interestingly, minor infiltrates can be seen in normal female C57BL/6 mice at 16 weeks of age with 26% frequency (data not shown). At the clinical- disease phase at approximately 31 weeks of age, salivary glands of both male and female C57BL/6.NOD-*Aec1Aec2* mice were positive for lymphocytic foci with 100% frequency (data not shown). Area analysis using regions of interest, illustrated in Fig. 2, revealed that female C57BL/6.NOD-*Aec1Aec2* developed larger infiltrates at 16 weeks of age in comparison to female C57BL/6. At 31 weeks of age, both male and female C57BL/6.NOD-*Aec1Aec2* mice developed similar size infiltrates but three to four times larger in area compared to both sexes of C57BL/6 mice. These data suggest that severe lymphocytic infiltration or sialadenitis occurs earlier in female C57BL/6.NOD-*Aec1Aec2* mice than the male counterpart during the adaptive phase with progressive severity during the clinical-disease phase.

### Severity of glandular infiltration in female C57BL/6.NOD-Aec1Aec2 mice is mediated by increased numbers of tissue-specific B and T lymphocytes

**Fig. 3.**
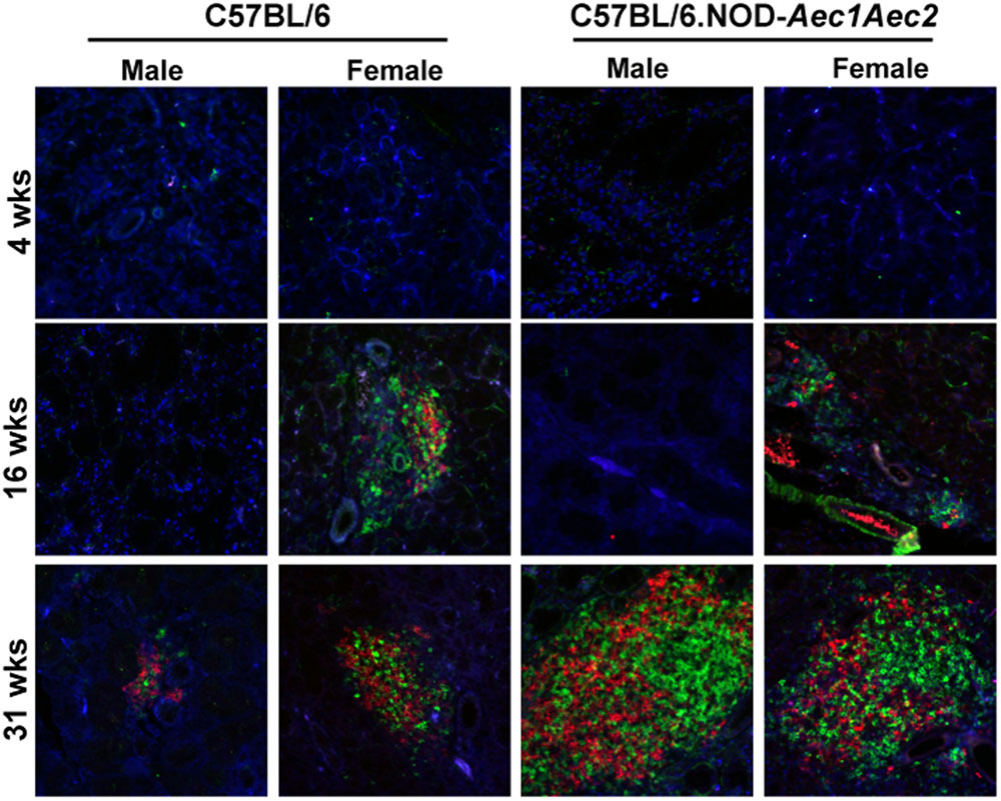
**Presence of salivary glands-specific B and T cells.** Male and female C57BL/6J.NOD-*Aec1Aec2* at 4, 16 and 31 weeks old (males, *n*=4, 4 and 6; females, *n*=3, 3 and 9 for three ages respectively) and male and female C57BL/6 at 4, 16 and 31 weeks of age (males, *n*=7, 6 and 5; females, *n*=3, 6 and 8 for three ages respectively) were used. Salivary glands were excised and embedded in paraffin. Sections were cut and stained for CD3^+^ T cells [donkey anti-goat FITC against goat anti-mouse CD3 (green)] and B220^+^ B cells [donkey anti-rat IgG AF594 against rat anti-mouse (red)] with DAPI nuclei staining (blue). Images were captured on Nikon Ti-E fluorescent microscope. Representative images were shown at 200× magnification.

**Fig. 4.**
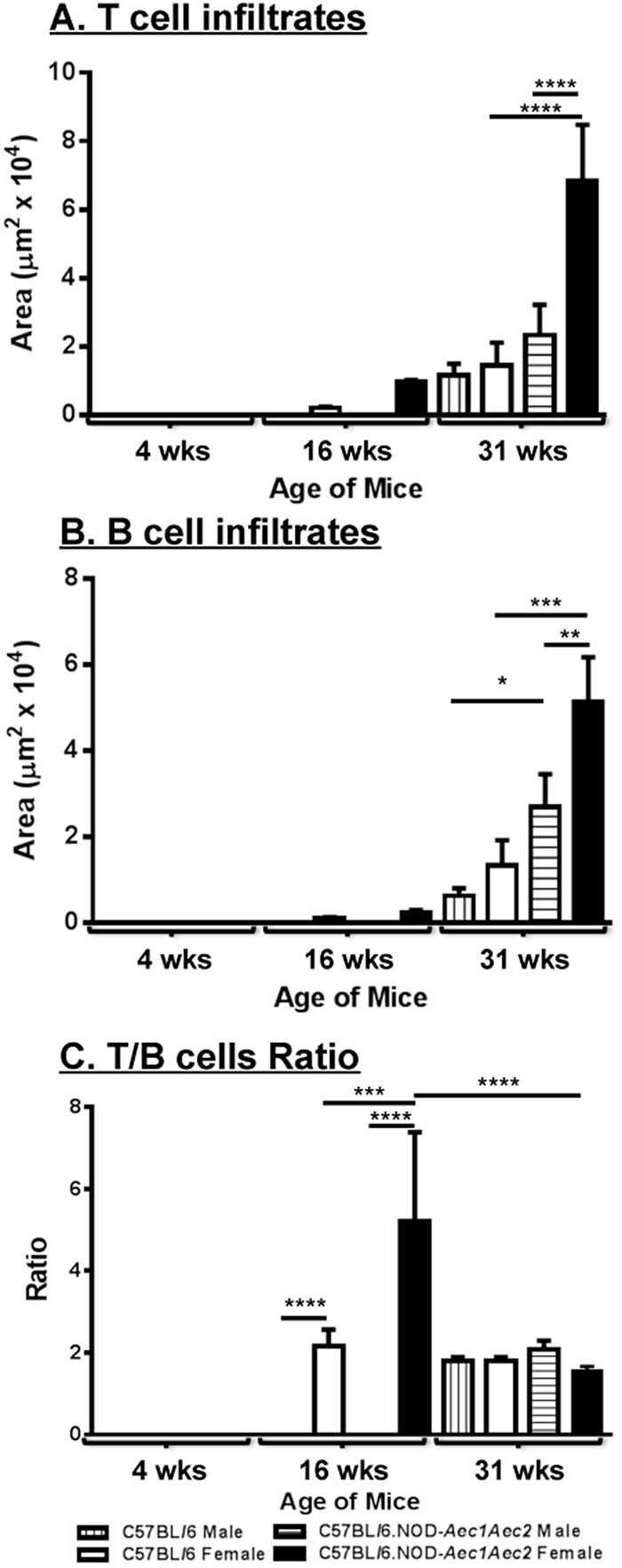
**Degree of CD3^+^ T cells and B220^+^ B cells infiltration in salivary glands.** Based on immunofluorescent staining for CD3/B220 in male and female C57BL/6 and C57BL/6J.NOD-*Aec1Aec2* presented in Fig. 3, measurement of areas using densitometrical analysis was calculated using Nikon Elements software with the percentage of threshold events in ROI function. The mean±s.e.m. was calculated for both the B and T cells (A,B), whereas the mean of the ratio of T:B cells was calculated (C); the significance for each was calculated by two-way ANOVA. Error bars indicate s.e.m. with *P<0.05, **P<0.01, ***P<0.001, ****P<0.0001.

The severity of glandular infiltration is enumerated based on the grade of gland histological lesions. Our studies using human SjS labial lip biopsies have indicated that small lesions appear to consist mostly of CD3^+^ T cells with larger foci occupied by a higher number of CD20^+^ B cells than CD3^+^ T cells. Larger histological lesions are indicative of disease severity, longer duration and more advanced clinical symptoms. In our animal model of SjS, similar observations were found in which early infiltrations are composed mainly of CD3^+^ T cells with equal number of CD3^+^T and B220^+^ B cells during the adaptive stage and higher number of B220^+^ B cells compared to CD3^+^ T cells at the clinical-disease stage. However, there is no data, which compares the changes within lymphocytic composition, specifically the temporal change over the course of the autoimmune reaction, in male and female human patients or animal models of SjS. As presented in Fig. 3, histological sections of male and female C57BL/6.NOD-*Aec1Aec2* and C57BL/6 mice were stained and examined for B220^+^ B cells and CD3^+^ T cells at 4, 16 and 31 weeks of age. As expected, no B220^+^ B cells and CD3^+^ T cells were detected at 4 weeks of age. Female C57BL/6.NOD-*Aec1Aec2* developed sialadenitis at 16 weeks of age with a higher number of B220^+^ B cells and CD3^+^ T cells compared to female C57BL/6 and none were detected in male C57BL/6.NOD-*Aec1Aec2* mice. At 31 weeks of age, both male and female C57BL/6.NOD-*Aec1Aec2* mice showed a significant increase in B220^+^ B cells and CD3^+^ T cells in comparison to C57BL/6 mice. Interestingly, as presented in Fig. 4A and B, densitometrical analyses revealed that female C57BL/6.NOD-*Aec1Aec2* mice exhibited a marked increase in both B220^+^ B cells and CD3^+^ T cells in comparison to male C57BL/6.NOD-*Aec1Aec2* mice at the adaptive and clinical-disease stages. Whereas all of the other strains exhibited a ratio of approximately 2:1 (T/B cells), female C57BL/6.NOD-*Aec1Aec2* mice at 16 weeks displayed at least five times the area of T cells composing these infiltrates (Fig. 4C). These data indicate female C57BL/6.NOD-*Aec1Aec2* mice develop earlier inflammatory lesions in the glands with a significantly higher number of B220^+^ B cells and CD3^+^ T in the later stages of SjS. Moreover, the significant decrease of T/B ratios from 16 weeks to 31 weeks of age by female C57BL/6.NOD-*Aec1Aec2* mice indicate a marked change in dominant T cells infiltration at pre-clinical disease to elevated number of B cells at the clinical disease which clearly mimics the human disease progression (Nguyen et al., 2008).

### Elevated salivary gland Th17 cells in female compared to male C57BL/6.NOD-Aec1Aec2 mice

**Fig. 5.**
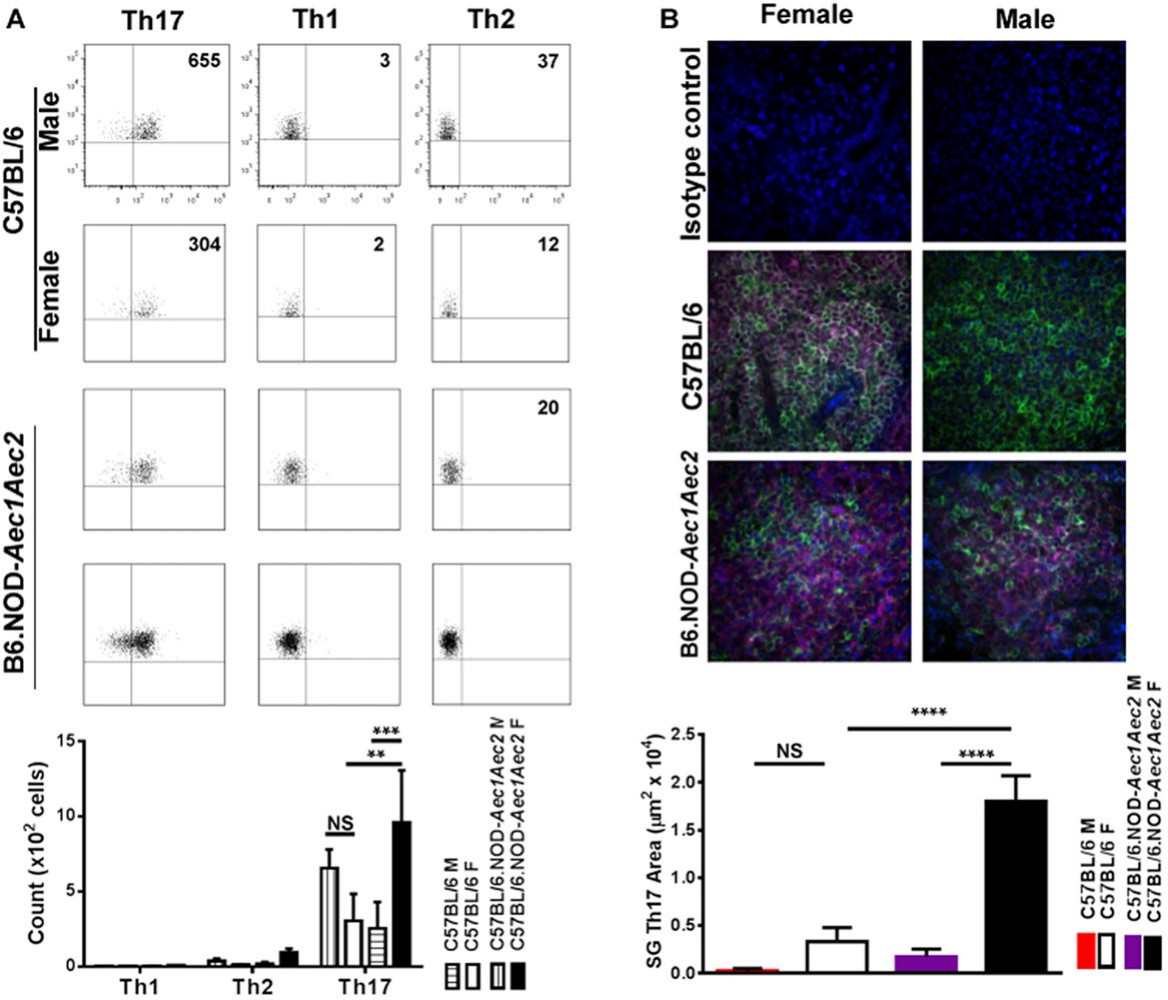
**Frequency of Th17 cells in salivary glands at clinical-disease stage.** Glands were excised and single-cell suspensions were isolated from male and female C57BL/6 and C57BL/6J.NOD-*Aec1Aec2* mice (*n*=6/sex/strain, 24-33 weeks of age). Flow cytometric acquisition was performed by staining with Brilliant Violet 605 anti-mouse CD3, APC-Alexa Fluor 750 Rat anti-mouse CD4, Pacific Blue anti-mouse IFN-γ, PerCP/Cy5.5 anti-mouse IL-17A, and PE/Cy7 anti-mouse IL-4. Data was analyzed by FlowJo software. Results shown are representative data with cell count numbers presented. Average cell counts with standard of error bars for Th1, Th2, and Th17 cells of each group were presented in the bar graph (A). Immunofluorescent staining for CD4 and IL-17 were performed on paraffin-embedded sections of the salivary glands of male and female C57BL/6 and C57BL/6J.NOD-*Aec1Aec2* mice (*n*=6/sex/strain, 24-33 weeks of age). Negative controls were performed with rabbit IgG isotype. Representative images were presented at 200× magnification (B). Area of salivary gland Th17 cell was enumerated using binary densitometrical analysis on Nikon Elements software. (CD4^+^, AF488-green; IL-17A^+^, AF647-pink; DAPI, blue) (B). The significance for each was calculated by two-way ANOVA. Error bars indicates s.e.m. with **P<0.01, ***P<0.001, ****P<0.0001.

We have found that Th17 cells and IL-17 levels are highly upregulated in the salivary glands of both male and female C57BL/6.NOD-*Aec1Aec2* mice with similar observation in patients (Nguyen et al., 2008). However, the comparative frequency of Th17 cells between the two sexes has never been examined during that time of clinical-disease with the most severe inflammation. Therefore, to determine the differential frequency of Th17 cells in the salivary glands of male and female mice after onset of the clinical signs, flow cytometric analysis of Th17 cells was performed at 24-33 weeks of age in which lymphocytic infiltration has occurred. As presented in Fig. 5A, female C57BL/6.NOD-*Aec1Aec2* mice exhibited a 4-fold increase in the number of Th17 cells compared to male disease mice. Interestingly, male C57BL/6 mice showed a higher frequency of Th17 cells in the salivary glands in comparison to the female counterpart. No significant differences of Th1 and Th2 cells were detected in male and female of normal and disease mice. To further confirm the prevalence of Th17 cells of female disease mice and to illustrate their presence in the glands, a histological analysis of IL-17^+^Th17 cells was performed. As illustrated in Fig. 5B, male and female control C57BL/6 mice developed smaller infiltrates in the salivary glands with less CD4^+^IL17^+^ cells at these ages. Histological examination of male and female C57BL/6.NOD-*Aec1Aec2* mice revealed severe lymphocytic infiltrations in the salivary glands and significant CD4^+^IL17^+^ cell infiltration was observed. Enumeration of total area per focus occupied by CD4^+^IL17^+^ cells showed that female C57BL/6.NOD-*Aec1Aec2* contained more than 10 times the area of male mice. These data demonstrate that Th17 and not Th1 or Th2 cells are the dominant cell population that makes up the lesions. There is a significant increase in Th17 cells in the salivary glands of female mice at the onset of clinical signs in the SjS animal model as compared to male mice.

### Hyperproliferation of autoreactive lymphocytes in salivary glands is inherent to female C57BL/6.NOD-Aec1Aec2 mice

**Fig. 6.**
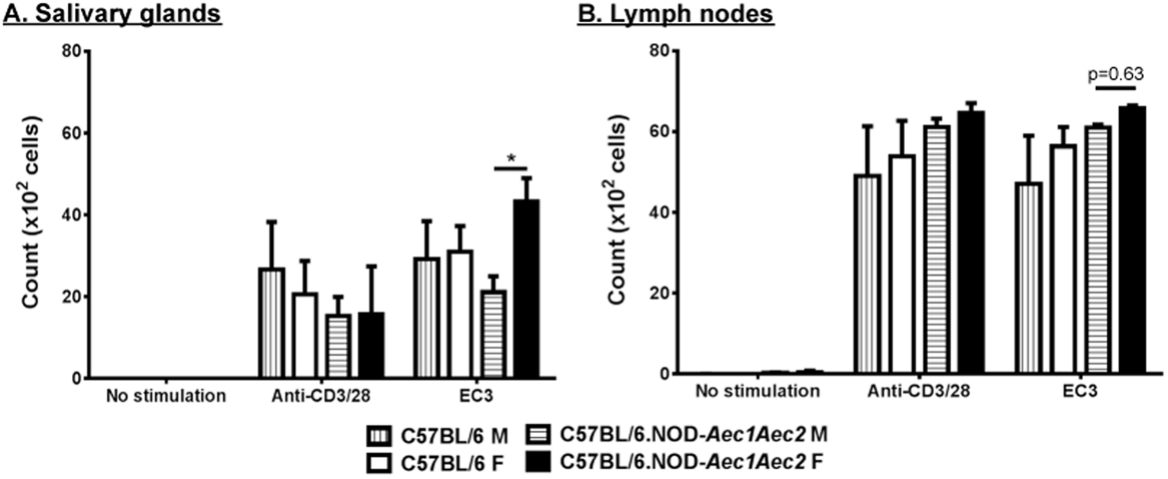
**Antigen-specific hyperproliferation of Th17 cells in female C57BL/6.NOD-*Aec1Aec2***. CFDA proliferation assay of salivary gland lymphocytes (A) and cervical lymph nodes (B) utilizing an antigen-non-specific manner (anti-CD3/anti-CD28), an antigen-specific activation (EC3, the third cytoplasmic loop peptide of M3R) and media alone as no stimulation for male and female C57BL/6 and C57BL/6.NOD-*Aec1Aec2*. Lymphocytes were incubated with CFDA prior to stimulation conditions (4 days), followed by flow cytometry and analysis on FlowJo software. Th17 cells were gated for live CD3^+^CD4^+^IL-17^+^. Statistically significant results were calculated with two-way ANOVA on male (*n*=5) and female (*n*=5) C57BL/6, and male (*n*=5) and female (*n*=3) C57BL/6.NOD-*Aec1/2* female. Error bars indicates s.e.m. with ****P<0.00001.

As demonstrated previously, gland lymphocytic infiltrates were detected at 16 weeks of age with more severe sialadenitis found in female C57BL/6.NOD-*Aec1Aec2* mice. The activation of adhesion molecules and chemokines may play a role in homing the immune cells to the glands. The underlying mechanism that facilitates immune cells to specifically target and invade the glands remains unknown. One fundamental feature of autoreactive lymphocytic cells in the glands is the hyperproliferation following stimulation in an antigen-specific and antigen-non-specific manner. To examine this intrinsic property of autoreactive lymphocytes in SjS, lymphocytes of lymph node and salivary gland cells were isolated from male and female C57BL/6 and C57BL/6.NOD-*Aec1Aec2* mice at 31 weeks of age (clinical symptoms). Cells were labeled with CFDA and stimulated with anti-CD3/anti-CD28 (antigen-non-specific activation) and the third cytoplasmic loop peptide of M3R (antigen-specific activation). CFDA-labeled proteins are inherited by daughter cells after cell division; therefore, the loss of fluorescent intensity by flow cytometric analysis is indicative of cell proliferation. As presented in Fig. 6, Th17 cells (CD3^+^CD4^+^IL17^+^) of lymph nodes from male and female C57BL/6 and C57BL/6.NOD-*Aec1Aec2* mice responded equally with anti-CD3/anti-CD28. Interestingly, Th17 cells in salivary glands of female C57BL/6.NOD-*Aec1Aec2* mice exhibited higher proliferation with M3R peptide stimulation compared to male C57BL/6.NOD-*Aec1Aec2* mice and both sexes of control mice (Fig. 6A). Similar results were found in the lymph nodes in which Th17 cells of female C57BL/6.NOD-*Aec1Aec2* mice elicited a trend of proliferative activity in comparison to male C57BL/6.NOD-*Aec1Aec2* mice when activated by M3R peptide (Fig. 6B). Minimal response was detected by non-Th17 cells (data not show). These data suggest that cervical draining lymph nodes and salivary glands contain mostly autoreactive Th17 cells that are specifically responsive against M3R autoantigen; however, a ‘female-milieu’ of C57BL/6.NOD-*Aec1Aec2* genetic background appears to enhance Th17 cell proliferation at the clinical-disease stage.

### Altered ANA patterns in female C57BL/6.NOD-Aec1Aec2 mice starting at the adaptive phase

**Fig. 7.**
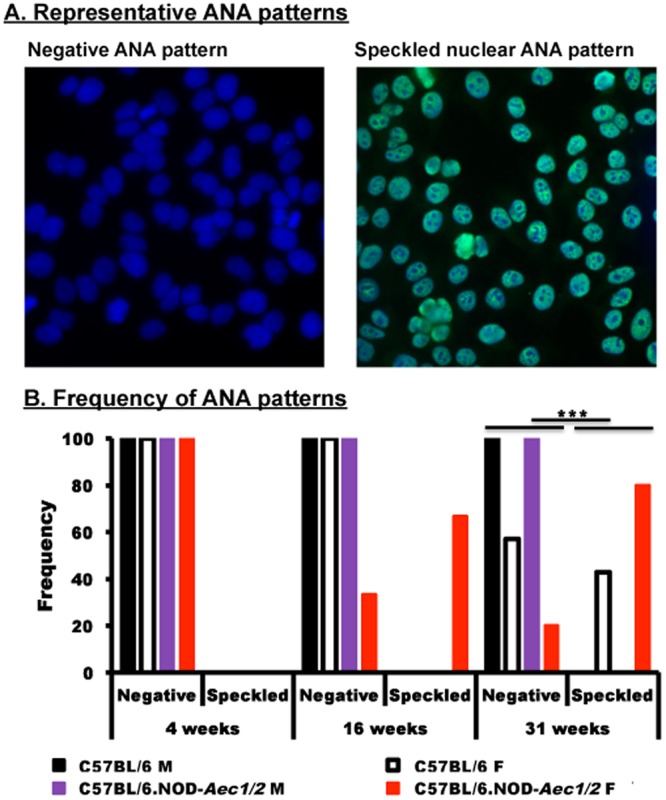
**Profile of anti-nuclear autoantibody patterns.** Sera of male and female C57BL/6 and C57BL/6J.NOD-*Aec1Aec2* mice was incubated with HEp2 cells, followed by incubation with AF 488 goat anti-mouse IgG (green) and set with VectorShield containing DAPI (blue) with at least *n*=6 per sex per strain per age group. Examples of negative and speckled staining patterns are given at 100× magnification (A). The bar graph displays the frequency of animals which showed the different staining patterns (B). The experiment was repeated twice. A Chi squared test was performed, where ***P<0.00001.

The appearance of B and T lymphocytes within the salivary glands of female C57BL/6.NOD-*Aec1Aec2* mice at 16 weeks of age, plus the significant increase in autoreactive lymphocyte proliferation on activation results in the elevated production of circulating autoantibodies, specifically ANA. To examine the levels and change in ANA patterns, staining of HEp-2 cells was examined using sera from male and female C57BL/6.NOD-*Aec1Aec2* and C57BL/6 mice at three disease phases. As presented in Fig. 7, the sera collected from both sex-matched strains at 4 weeks of age were negative for ANA. At 16 weeks of age, 70% of female C57BL/6.NOD-*Aec1Aec2* mice shifted to speckled nuclear profile while none of the other strains exhibited similar changes. At 31 weeks of age, female C57BL/6.NOD-*Aec1Aec2* mice showed a slight increase in speckled nuclear profile from 16 weeks of age. Interestingly, female C57BL/6 showed a 25% shift to a speckled nuclear pattern at 31 weeks of age. The shift in the ANA pattern with the changes in influx and proliferation of lymphocytes in the salivary glands of female C57BL/6.NOD-*Aec1Aec2* mice at 16 weeks of age supports the concept that a number of immunological responses initiate during the adaptive immune response in order to set up the overt clinical disease pathology at later ages.

### Rapid loss of secretory function during the adaptive phase in female C57BL/6.NOD-Aec1Aec2 mice

**Fig. 8.**
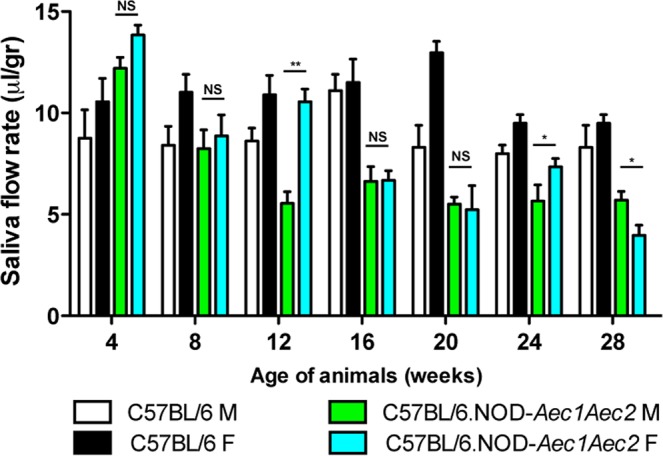
**The onset of salivary secretory dysfunction.** Saliva flows were collected as described in the Materials and Methods section. The data shown represent the mean saliva flow rate (volume/weight or μl/g)±s.e.m. per group (*n*=8, female C57BL/6, *n*=7 male C57BL/6, *n*=7 female C57BL/6.NOD-*Aec1Aec2* female, *n*=8 male C57BL/6.NOD-*Aec1Aec2*). The statistical differences between male and female C57BL/6.NOD-*Aec1Aec2* were determined using one-tailed Mann–Whitney tests at all threshold events. Error bars indicate s.e.m. with *P<0.05, **P<0.01, NS, no significant.

Secretory dysfunction could be contributed by acinar cell death mediated by the intrinsic nature of the autoimmune-susceptible background or an inflammatory response initiated from the glands and/or peripheral lymphoid organs. As indicated in Fig. 8, both male and female C57BL/6.NOD-*Aec1Aec2* mice exhibited initial loss of saliva secretion at 8 weeks of age with males showing earlier elevated loss at 12 weeks of age. Significant secretory dysfunction was observed at 16 weeks of age and continued at subsequent time points. Interestingly, during this time frame male C57BL/6.NOD-*Aec1Aec2* mice showed a gradual decrease with a 70% loss of saliva secretion in a 16-week span (4 weeks-20 weeks), while female C57BL/6.NOD-*Aec1Aec2* mice displayed a rapid loss of 70% within a 8-week period (12 weeks-20 weeks). C57BL/6 mice appeared to fluctuate saliva secretion at similar time frame with a higher contrast at 20 weeks of age; however there were no overall significant differences in male and female mice. The data provide a clear indication that although male C57BL/6.NOD-*Aec1Aec2* mice appear to exhibit an early onset of saliva loss, female C57BL/6.NOD-*Aec1Aec2* mice show a more rapid loss of secretory function once the disease is established.

## DISCUSSION

SjS is a systemic autoimmune disease with one of the highest female to male disparities. The precise biological mechanism governing this sexual dimorphism remains elusive. One of the major challenges is studying the covert autoimmune process of the disease prior to the overt clinical disease. The other challenge is the lack of male cohorts to perform a proper and essential comparative analysis between male and female patients. As a result, identifying an appropriate animal model will allow us to examine both the covert and overt autoimmune process, and more importantly better facilitate the comparison between males and females. Using the C57BL/6.NOD-*Aec1Aec2* animal model, we have shown that female C57BL/6.NOD-*Aec1Aec2* mice developed an earlier and more severe onset of sialadenitis. Infiltrates were composed of a significantly higher number of CD3^+^T cells with a 10-fold increase in glandular infiltration of CD4^+^IL17^+^ Th17 cells in female compared to male mice at the onset of the clinical disease. In addition, gland specific Th17 cells exhibited a robust proliferative capacity in female C57BL/6.NOD-*Aec1Aec2* mice. Infiltrated B cells were significantly elevated in females compared to males with a concomitant altered ANA pattern observed solely in female C57BL/6.NOD-*Aec1Aec2* mice. Secretory dysfunction was discerned in both sexes in which females developed a later and more rapid onset of secretory dysfunction once the disease is established. This data indicates significant differences at various stages of the autoimmune process in males and females, suggesting a clear sexual dimorphism in the C57BL/6.NOD-*Aec1Aec2* animal model.

SjS development is attributed to a number of essential factors; for examples genetic-susceptible background, suitable environments like sex hormones, and dysregulation of immune cell activation (Bolstad and Jonsson, 2002; Mostafa et al., 2012; Porola et al., 2007). These same factors can also impose the sexual dimorphic development of the disease. The NOD mouse has also become a popular animal model for SjS-like disease, in part due to its many similarities with human SjS, ranging from decreased secretory function to the presence of lymphocytic infiltrates in the exocrine glands (Hu et al., 1992; Humphreys-Beher et al., 1993, 1994; Robinson et al., 1996). Other NOD-derived animal models, specifically NOD.B10-*H2^b^* and NOD.H2^h4^, have been used to examine the role of MHC and non-MHC association in SjS. Sex differences were observed in female NOD.H2^h4^ mice in which female mice developed increased severity of sialadenitis with higher CD4^+^ T cells producing Th2 and Th17 cytokines. Females developed enhanced B cell responses with higher levels of serum anti-SSA, anti-SSB and B cell activation factor (Čiháková et al., 2009). Our data indicate an earlier onset of sialadenitis in female C57BL/6.NOD-*Aec1Aec2* mice with a higher number of T cells compared to B cells at the adaptive disease phase which is followed by an increase in infiltrating B cells at the clinical disease stage. Remarkably, the inflammatory lesions are composed primarily of Th17 cells at the clinical disease stage where female C57BL/6.NOD-*Aec1Aec2* mice showed a 10-fold increase in Th17 cells to males. This observation is consistent with the finding in human patients in which elevated CD20+ B cells with increased in Th17 cells can be seen in sera and labial lip biopsies. The data supports the current paradigm defining initial salivary gland infiltrates are T cells, carrying out the cell destruction and producing pathogenic cytokines and chemokines to recruit additional autoreactive B cells. Autoantibodies produced by these autoreactive B cells are one of the main culprits in SjS secretory dysfunction in both mouse and human of SjS. This study indicates altered ANA pattern at 31 weeks of age in female C57BL/6.NOD-*Aec1Aec2* mice, suggesting a shift to a more pathogenic antibody profile. Further studies are needed to examine the role of Th17 cells at the clinical-disease stage as to whether they function as helper T cells by promoting antibody production, or serve as glandular destructive cells, or their presence serves as homing signal to other cells to execute autoimmune responses.

Genetics play a significant role in SjS and other autoimmune diseases. In addition, environmental factors such as sex hormones are also involved, possibly by modulating the immune response. An earlier study (Hunger et al., 1998) showed that the NOD model exhibited sex dimorphic pathology between lacrimal and salivary glands in which male mice developed a significant infiltration in lacrimal glands at 12 weeks of age whereas age-matched female NOD mice lacked local inflammation. Orchidectomized NOD males showed a decrease in dacryoadenitis with an increase in submandibulitis similar to NOD females. However, the ovariectomy of female NOD mice had little affect on exocrine gland infiltration. Fine mapping of the *idd3* (*Aec1*) region of NOD-derived C57BL/6.NOD-*Aec1Aec2* mice revealed that a sub-region between 2.4 and 19.2 cM is associated with stomatitis sicca in the absence of detectable keratoconjunctivitis sicca in female mice, whereas males maintained the full SjS disease (Nguyen et al., 2006b). These data suggest that the SjS disease in the NOD mouse shows gender-specific regulation determined by autosomal genes.

Having the susceptible genetic background that synergistically interacts with changing hormonal levels can enhance and possibly result in autoimmunity. McClellan et al. (2014) have shown that whereas aging C57BL/6 mice exhibited susceptibility for dry eye disease with more of a Th1 response developed in male mice, females were predisposed to Th17 bias in the conjunctiva. Our previous studies (Delaleu et al., 2013; Killedar et al., 2006) have indicated that prior to activation of innate and adaptive immune responses, perturbations in the salivary gland homeostasis and integrity can be observed; specifically the alterations in the epithelial cell–extracellular matrix (ECM) anchored focal adhesions (FAs), deceleration of ECM turnover and down regulation of genes encoding gap junction proteins. It is unknown what specific pathophysiological changes occur in the normal C57BL/6 background; however, our data indicate an influx of glandular lymphocytes at 16 weeks of age in females with an incremental increases in Th17 cells that are not antigen specific. It is possible that the additive effect of susceptible genetic background with SjS promoting autosomal genes directly contribute to the earlier onset and severity observed in female C57BL/6.NOD-*Aec1Aec2* mice.

During the adaptive immune response, there is an increase in the chemokine receptor-ligand profile characterized emigration of multiple APC and lymphocyte populations, as well as recruitment of Th1 cells, Th17, and NK cells (Janeway et al., 2001). Although estrogen has very little effect on glandular ECM, it has been shown to be anti-apoptotic in salivary gland cells (Konttinen et al., 2012). More importantly, there is strong evidence, which suggests that estrogen can greatly enhance the levels of IL-17 and Th17 cells by activation of its Rorγt transcription factor (Khan et al., 2010). Therefore, it is possible that the increase in the severity of sialadenitis and inflammatory Th17 cells in female glands is attributed to endogenous estrogen function. In addition, hyperproliferation of lymphocytes with the concomitant presence of autoantibodies are classic hallmarks of SjS. At the clinical stage, our data has shown that female C57BL/6.NOD-*Aec1Aec2* mice exhibited an increase in antigen-specific Th17 cell infiltration accompanied by the change in ANA profiles and proliferative capacity. This drastic change in autoreactive Th17 cell function could be attributed to activity of sex hormones, particularly estrogen. High levels of estrogen can result in an increase in Th17 cells as discussed (Khan et al., 2010). Additionally, estrogen increases the number of Th2 cells producing IL-4, thereby leading to the activation of B cells to produce high levels of immunoglobulins and autoantibodies (Oertelt-Prigione, 2012). Other studies indicated that estrogen milieu can promote marginal zone B cells to produce autoreactive anti-dsDNA (Grimaldi et al., 2002; Jeganathan et al., 2014; Venkatesh et al., 2006), therefore it is not surprising that female sex hormones can modulate specific T cell subsets such as Th17 cells observed in this study.

The impact of androgen on the immune response is still controversial but generally it is thought to exert suppressive potential (Cutolo et al., 2002; Fijak et al., 2011). Studies have indicated that like estrogen, androgen has an important role in preventing apoptosis of gland epithelium and in SjS glands (Konttinen et al., 2012; Spaan et al., 2009). When androgen regulation is defective, acinar atrophy and ductal cell hyperplasia can occur (Porola et al., 2010). The multifactorial cause of androgen function and androgen defective signaling on both gland epithelial and immune cells could establish a receptive condition for early loss of secretory function seen in male C57BL/6.NOD-*Aec1Aec2* mice. Alternatively, the loss of saliva at the later stage of the disease in male C57BL/6.NOD-*Aec1Aec2* mice could be attributed to the inhibition of IFN-γ-producing Th1 cells by androgen, which favors a Th2 response. Changing to a Th2 response could potentially contribute to the emergence of circulating autoantibodies that are undetected at this stage, independent of glandular pathological changes (Dawson et al., 2006). Interestingly, female C57BL/6.NOD-*Aec1Aec2* mice showed a rapid ∼50% loss of saliva from 12 weeks to 16 weeks of age. Again, this data clearly indicates the activation of the adaptive immune response with more hyperactive and antigen-specific Th17 cells resulting in pathogenic speckled autoantibody profiles (such as anti-Ro, anti-La and anti-M3R). The potent activation of autoreactive B and T cells, which were found to be highly upregulated at the clinical-disease stage might contribute to a more rapid loss of salivary gland function in female C57BL/6.NOD-*Aec1Aec2* mice.

The current study has demonstrated that C57BL/6.NOD-*Aec1Aec2* mice exhibited sexual dimorphic disease development. There are a number of factors that require further investigation. The focus of this study is limited to the salivary gland function; however, lacrimal gland function is the other key manifestation of SjS. Additional work is needed to completely profile the possible sexual dimorphism in the lacrimal glands. Moreover, the precise stage of the disease in which estrogen and androgen are involved needs to be defined. Presently, it is thought that sex hormones have a limited role in glandular development; however, it is possible that defective estrogen/androgen signaling can perturb glandular development leading to the onset of SjS. We need to define the precise mechanism(s) on which sex hormones modulate the differentiation and function of autoreactive lymphocytes in particular pathogenic Th17 cells and autoantibody-producing B cells. Lastly, the immune regulatory component mediated by sex hormones on immune cells and salivary gland epithelium needs to be examined to fully understand the autoimmune process of SjS.

## MATERIALS AND METHODS

### Animals

SjS^s^ C57BL/6J.NOD/ShiLtJ-*Aec1Aec2* (C57BL/6.NOD-*Aec1Aec2*) and non-SjS^s^ C57BL/6J (C57BL/6) control mice were bred and maintained under specific pathogen free conditions in the animal facility of Animal Care Services at the University of Florida. The breeding and the use of animals described herein were approved by the University of Florida's Institutional Animal Care and Use Committee. Development of the C57BL/6.NOD-*Aec1Aec2* mouse and its SjS-like disease phenotype are described elsewhere (Brayer et al., 2000; Cha et al., 2002b). Briefly, the C57BL/6.NOD-*Aec1Aec2* mouse was developed by introducing two genetic regions, one on chromosome 1 (designated *Aec2*) and the second on chromosome 3 (designated *Aec1*) derived from the NOD/LtJ mouse into the C57BL/6 mouse. All animals were maintained on a 12 h light-dark schedule and provided food and acidified water *ad libitum*. At times indicated in the study, mice were euthanized by cervical dislocation after deep anesthetization with isoflurane and their organs and tissues freshly harvested for analyses.

### Histological examination of the salivary glands

Salivary glands were fixed in 10% phosphate-buffered formalin for 24 h. Fixed tissues were embedded in paraffin and sectioned at a thickness of 5 μm. Paraffin-embedded sections were deparaffinized by immersing in xylene, followed by dehydration in ethanol. The prepared tissue sections were stained with hematoxylin and eosin (H&E) dye (Histology Tech Services, Gainesville, FL). Stained sections were observed at 200× magnification by using Nikon Eclipse Ti-E inverted microscope. To detect and determine leukocytic infiltrations in salivary glands, a single histological section per gland per mouse was examined by a blinded examiner. Lymphocytic infiltrations were defined as aggregates of >50 leukocytes.

### Immunofluorescent staining for CD3+T cells and B220+B cells

Paraffin-embedded tissues of the salivary glands were sectioned and mounted onto microscope slides. Slides were deparaffinized and dehydrated by pressure-cooking in Trilogy (Cell Marque, Rocklin, CA) according to manufacturer's instructions. Following three 5-min washes with phosphate buffered saline with Tween-20 (PBS-T) at 25°C, the sections were incubated one hour with blocking solution containing donkey serum diluted 1:50 in PBS-T. Each section was incubated with purified rat anti-mouse CD45R (Clone 30-F11, BD Pharmingen, San Jose, CA, USA) diluted 1:25 and goat polyclonal IgG anti-mouse CD3ε (Clone M-20, Santa Cruz Biotechnology, Santa Cruz, CA, USA) diluted 1:50 in antibody diluent (Dako, Carpinteria, CA, USA) for one hour at 25°C. The slides were washed three times with PBS-T, followed by a one hour incubation with Alexa Fluor (AF) 488 donkey anti-goat IgG (H+L) diluted 1:100 and AF 594 donkey anti-rat IgG (H+L) (Life Technologies, Grand Island, NY, USA) diluted 1:25 at 25°C. The slides were washed thoroughly with PBS-T, treated with VectashieldDAPI (4′,6-diamidino-2-phenylindole)-mounting medium (Vector Laboratories, Burlingame, CA, USA) and overlaid with glass coverslips. Stained sections were visualized at 200× magnification on NikonTi-E fluorescent microscope. Infiltrate size and composition were calculated using Nikon NIS-Elements software by a blinded examiner. Areas of infiltrates were determined using the region of interest (ROI) established around the infiltrates and the composition based on the threshold intensities with background subtraction for individual fluorescent channels. The threshold intensities were kept consistent throughout the experiment.

### Examination of salivary gland T helper cells by flow cytometry and immunofluorescent staining

Salivary glands were digested in a digest buffer (1 mg/ml DNase (Sigma-Aldrich, St. Louis, MO, USA) and 1 mg/ml Collagenase Type 4 (Worthington, Lakewood, NJ, USA) in RPMI (Lonza, Allendale, NJ, USA) complete media containing 10% FBS, 2 mM L-glutamine, 0.05 mM β-mercaptoethanol) and placed in a MACS C tube (Miltenyi Biotec, San Diego, CA, USA) for desiccation on GentleMACS V1.02 for a pulse of 38 s, twice. After a 10-min incubation at 37°C the digest buffer was removed and placed into 4°C RPMI complete media and replaced with another digest buffer to repeat the process twice more. Single-cell suspensions were centrifuged (2500 rpm, 10 min, 4°C) and resuspended in PBS for filtration through a 70-μm sterile cell strainer (Fisher, Pittsburgh, PA, USA). After a wash with PBS, cells were resuspended again in PBS for lymphocyte isolation with Lympholyte-M cell separation media (Cedar Lane, Bulington, Ontario, Canada) per manufacturer's instructions. Single-cell suspensions were stained for surface markers, Brilliant Violet 605 anti-mouse CD3 (Clone 17A2, Biolegend, San Diego, CA, USA) and APC-Alexa Fluor 750 Rat anti-mouse CD4 (Clone RM4-5, Invitrogen, Grand Island, NY, USA). The membranes were permeabilized using Cytofix/Cytoperm Fixation/Permeabilization kit and intracellularly stained for Pacific Blue anti-mouse IFN-γ (Clone XMG1.2, eBioscience, San Diego, CA, USA), PerCP/Cy5.5 anti-mouse IL-17A (Clone TC11-18H10.1, Biolegend), and PE/Cy7 anti-mouse IL-4 (Clone 11B11, Biolegend) per manufacturer's instruction (BD, Franklin Lakes, NJ, USA). The samples were analyzed using BD Fortessa Flow Cytometer (BD Biosciences, San Jose, CA, USA) and analysis was performed using FlowJo VX software (FlowJo, Ashland, OR, USA). Immunofluorescent staining on paraffin-embedded tissues of the salivary glands were previously described (Nguyen et al., 2006a). In brief, slides were de-paraffinized and each section was incubated with purified goat anti-mouse CD4 diluted 1:25 and rabbit polyclonal IgG anti-mouse IL-17 (Santa Cruz Biotechnology) diluted 1:25 in antibody diluent (Dako) for one hour at 25°C. The slides were incubated with AF 488 donkey anti-goat IgG (H+L) diluted 1:100 and AF647 donkey anti-rabbit IgG (H+L) (Life Technologies) diluted 1:25 at 25°C. Slides were treated with Vectashield DAPI (4′,6-diamidino-2-phenylindole)-mounting medium (Vector Laboratories, Burlingame, CA, USA) and overlaid with glass coverslips. Stained sections were visualized at 200× magnification on Nikon Ti-E fluorescent microscope. Infiltrate size and composition were calculated using Nikon NIS-Elements software; ROI was established around the infiltrate and the composition, based on the threshold intensities and backgrounds for the individual channels, was determined. The CD4^+^ T cells were selected first, followed by the IL-17^+^ within the CD4^+^ positive region, providing the area of the double positive signal. The threshold intensities were kept consistent throughout the experiment.

### Carboxyfluoresceindiacetate (CFDA) proliferation assay

Single-cell suspensions of salivary glands were obtained as previously described (Robinson et al., 1998). After a wash with PBS, lymphocytes were tagged with ester via Vybrant CFDA SE Cell tracer kit (Life Technologies) by manufacturer's instruction. After incubation, cells were washed and plated with either media only (no stimulation), anti-CD3/anti-CD28 microbeads (T cell activation/expansion kit, Miltenyi Biotec), or 10 ng/ml M3R third extracellular loop peptide (EC3), NH2- VLV NTF CDS CIP KTY WNL GY -COOH (Biosynthesis Inc., Lewisville, TX, USA). After four days, cells were rinsed and resuspended in FACS buffer. The samples were analyzed using BD Fortessa Flow Cytometer (BD Biosciences) and analysis was performed using FlowJo VX software (FlowJo, Ashland, OR, USA).

### Detection of antinuclear antibodies (ANA) in the sera

Anti-nuclear antibodies were detected in the sera of mice by using HEp-2 ANA kit (Inova Diagnostics, Inc., San Diego, CA, USA). All procedures were performed according to the manufacturer's instructions. Mouse sera were diluted 1:80 and incubated on HEp-2-fixed substrate slides for one hour at room temperature in a humidified chamber. After three 5-min washes with PBS, the substrate slides were treated with a 1:100 dilution of AF 488 goat anti-mouse IgG (H+L) (Life Technologies) for 45 min at room temperature. After three washes, slides were treated with Vectashield DAPI-mounting medium (Vector Laboratories) and overlaid with glass coverslips. Fluorescence was detected by fluorescence microscopy at 400× magnification by using a Nikon microscope, and all images were obtained with exposure of 200 ms.

### Measurement of saliva flow

To measure stimulated flow rates of saliva, individual mice were weighed and given an intraperitoneal (ip) injection of 100 μl of a mixture containing isopreterenol (0.2 mg/1 ml of PBS) and pilocarpine (0.05 mg/1 ml of PBS). Saliva was collected for 10 min from the oral cavity of individual mice using a micropipette starting one minute after injection of the secretagogue. The volume of each saliva sample was measured.

### Statistical analyses

Statistical evaluations for sialadenitis, B and T infiltrates in the glands, Th17 cells and Th17 cell proliferation were determined by using two-way ANOVA. A Chi squared test was used for autoantibody patterns and one-tailed Mann–Whitney test was used for salivary function. Analyses were generated by the GraphPad Prism 6 software (GraphPad Software**,** La Jolla, CA, USA).
